# Surgical Correction of Posttraumatic Scapulothoracic Bursitis, Rhomboid Major Muscle Injury, Ipsilateral Glenohumeral Instability, and Headaches Resulting from Circus Acrobatic Maneuvers

**DOI:** 10.1155/2015/302850

**Published:** 2015-07-26

**Authors:** John G. Skedros, Tanner D. Langston, Colton M. Phippen

**Affiliations:** ^1^The University of Utah Department of Orthopaedic Surgery, Salt Lake City, UT 84108, USA; ^2^Utah Orthopaedic Specialists, Salt Lake City, UT 84107, USA; ^3^Intermountain Medical Center, Salt Lake City, UT 84157, USA

## Abstract

We report the case of a 28-year-old transgender (male-to-female) patient that had a partial tear of the rhomboid major tendon, scapulothoracic bursitis, and glenohumeral instability on the same side. These conditions resulted from traumatic events during circus acrobatic maneuvers. Additional aspects of this case that make it unique include (1) the main traumatic event occurred during a flagpole exercise, where the patient's trunk was suspended horizontally while a vertical pole was grasped with both hands, (2) headaches were associated with the periscapular injury and they improved after scapulothoracic bursectomy and rhomboid tendon repair, (3) surgical correction was done during the same operation with an open anterior capsular-labral reconstruction, open scapulothoracic bursectomy without bone resection, and rhomboid tendon repair, (4) a postoperative complication of tearing of the serratus anterior and rhomboid muscle attachments with recurrent scapulothoracic pain occurred from patient noncompliance, and (5) the postoperative complication was surgically corrected and ultimately resulted in an excellent outcome at the one-year final follow-up.

## 1. Introduction

Scapulothoracic bursitis is a condition that, when most advanced, is also known as snapping scapula syndrome. This condition can result from trauma to the scapulothoracic region and it is usually not associated with scapular winging [1–6]. Overuse, atraumatic, and traumatic glenohumeral instability, ranging from uni- to multidirectional, can also lead to scapular dyskinesia that can ultimately result in scapulothoracic bursitis with or without scapular winging [2, 3, 7–10]. But it is rare to have glenohumeral instability, a rhomboid major tear, and scapulothoracic bursitis resulting from the same traumatic event. The rarity of the association of these conditions as sequelae of trauma is reflected in our inability to locate more than a few reports describing patients with at least two of these conditions on the same side [2, 11].

We report the case of a 28 year-old patient that had these anterior and posterior injuries (without scapular winging) on the same side while performing acrobatic exercise maneuvers. The pathologies were corrected during the same surgery with an open anterior capsular-labral reconstruction, rhomboid tendon repair, and scapulothoracic bursectomy without bone resection. Of further interest in this case is that the patient also reported having new onset migraine headaches that were triggered by periscapular pain that was associated with the periscapular injury. These headaches were also relieved after surgery. But the periscapular pain (not the glenohumeral symptoms) and headaches recurred subsequent to unadvised physical activity six-to-eight weeks after surgery. Ten months later (one year after the index surgery) a revision operation was done in the same location of the prior scapulothoracic bursectomy and tendon repair. Operative findings included partial tearing of the origin of the serratus anterior and rhomboid major at the medial margin of the mid-scapula in addition to recurrent bursitis and scar tissue formation. Excision of the inflamed bursa and scar tissue and repair of the muscle attachments led to an excellent outcome at final follow-up one year later.

## 2. Case Report

**Figure 1:**
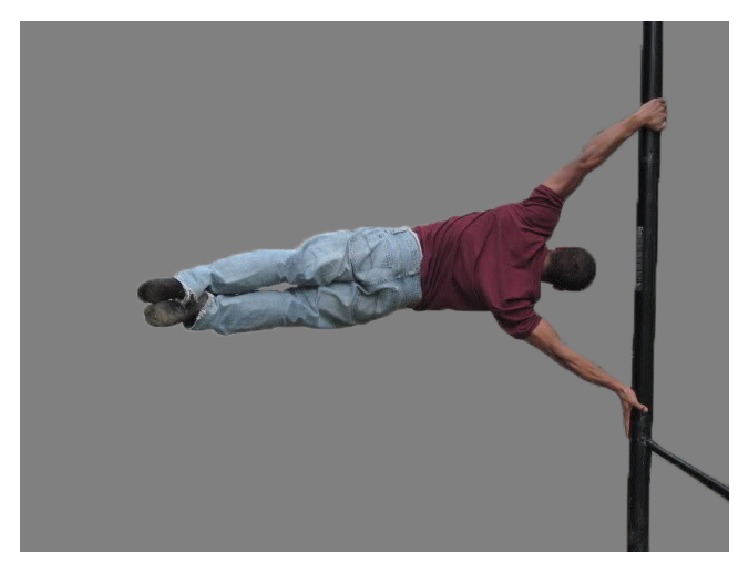
An example of the flagpole exercise.

A 28-year-old right-hand dominant transgender (male to female) patient (height: 180 cm; weight: 78 kg; BMI: 25) who was employed in a clerical occupation (and a previous part-time circus performer) presented to our clinic (April 5, 2012) with chief complaints of pain over the anterior and posterior aspects of the right shoulder and scapula regions and a feeling of shoulder instability (“it slips downward with forward and upward reaching”). The patient also reported painful “grinding and popping” beneath the mid portion of the medial aspect of the ipsilateral scapula. The patient attributed these symptoms to two traumatic events that occurred during circus acrobatic maneuvers. The first was during a “meat hook” maneuver on a “lyra,” which caused sudden pain over the medial aspect of the mid scapula on the right side. This improved over a two-month period. Four months later, the main traumatic episode occurred on the same side during “pole fitness exercises,” which was 15 months prior to the patient's first visit to our clinic. This main traumatic event occurred during a “flagpole” maneuver that placed the patient's body perpendicularly from a vertically oriented pole (Figure 1). The patient's right hand (the injured side) was grasping the pole below the lower extremities and trunk while the patient was suspended horizontal to the ground. Hence, the patient's right upper extremity was carrying most of the load. While performing this maneuver the patient's right shoulder “gave way,” dislocating and spontaneously relocating. The patient did not fall to the ground. The initial pain was greatest along the medial scapula; the instability of the shoulder region and the scapular-thoracic crepitus became significant several weeks later. There was no neck or head pain and no other chest wall injury. Chiropractic treatment was sought three weeks later. Significant improvement did not occur despite several months of treatment and isometric shoulder strengthening.

The patient was otherwise generally healthy except for being diagnosed with hemochromatosis one year earlier (H63D mutation of the HFE gene). As part of the pharmacological treatment for being transgender, the patient had been receiving estradiol valerate injections (10 mg intramuscular every other week) for two years prior to the shoulder girdle trauma. The only other medical problem was migraine headaches (1-2 per week), which were associated with visual and auditory auras [12]. These never occurred prior to the shoulder girdle injuries and they were triggered by activity-related increases in periscapular pain, and then the pain migrated to the posterior skull base and ultimately involved the patient's “entire head.”

Our first physical examination of the patient's right shoulder girdle (15 months after the trauma) showed a moderate sulcus sign and positive apprehension and relocation maneuvers that were consistent with anterior-inferior glenohumeral instability. There was also pain and crepitus over the mid-scapula with shoulder elevation with mild scapular dyskinesia suggestive of inferior trapezius weakness but without scapular winging. There was no palpable defect, no limitation of shoulder motion, and no Spurling's sign, neurological abnormalities, or evidence of generalized ligamentous laxity. Anterior (Grashey view), scapular-Y, and axillary-lateral radiographs of the right shoulder showed normal morphology and no evidence of a Hill-Sachs lesion. Radiographs of the scapula also showed normal morphology.

Because nonoperative measures had not been exhausted, the patient was referred to a physical therapist with orders to evaluate and treat glenohumeral instability and scapulothoracic bursitis using published protocols [13, 14]. Oral prednisone was also prescribed with instructions to follow a tapered schedule over 2.5 weeks (20 mg/day to 2.5 mg/day). This was aimed at reducing the scapulothoracic bursitis and pain in order to enhance progression with the patient's physical therapy [7, 13]. Referrals were also made to a spine specialist and a neurologist to evaluate and treat the patient's headaches and to rule out cervical spine pathology.

After a normal neurological examination was independently confirmed and magnetic resonance (MR) scans of the patient's brain and neck showed no abnormalities, the patient then returned to our clinic with persistent and more clearly defined signs and symptoms of glenohumeral instability and scapulothoracic bursitis. A MR scan with intra-articular contrast (arthrogram) of the right shoulder revealed tears of the anterior-superior labrum and middle glenohumeral ligament. A completed tomography (CT) scan of the right scapulothoracic regions revealed normal scapula morphology and no unusual tissue. A right scapulothoracic bursa shoulder injection was then done, which included 5 cc of 1% lidocaine, 5 cc of 0.25% bupivacaine, and 1.5 cc of 80 mg of methylprednisolone acetate. The purpose of the injection was threefold: (1) to attempt to improve the painful scapulothoracic bursitis without resorting to surgery [1, 13], (2) to determine if the headaches would be temporarily relieved, and (3) to determine what percentage of the total pain would be relieved as a result of the local anesthetic effect. The injection alleviated approximately 80% of the patient's shoulder girdle pain and reduced the crepitus for three weeks and the headaches stopped occurring for four weeks. By the fourth week after the injection there was recurrence of periscapular pain and crepitus and the headaches. However, because the crepitus was less, a second scapulothoracic injection with local anesthetic and corticosteroid was done four weeks later. But this did not provide sufficient lasting relief.

After failure of these nonoperative measures and after obtaining an opinion from an internal medicine physician that the patient's hemochromatosis and transgender status were not contributing to the patient's headaches and other symptoms, we recommended scapulothoracic bursectomy (the rhomboid major injury was not yet recognized) and capsulolabral reconstruction to be done during the same surgery. These procedures were performed by J. G. Skedros nine months after the patient's initial visit to our clinic.

For the surgery, the patient was placed in a lateral position with traction applied to the right upper extremity. The anterior labrum was found to be frayed and was arthroscopically debrided and an open capsule shift was performed [15]. The capsular shift was done with open technique because the patient desired to return to aggressive acrobatic exercise activities [16]. This portion of the procedure was done with the patient tilted 30 degrees in the posterior direction.

**Figure 2:**
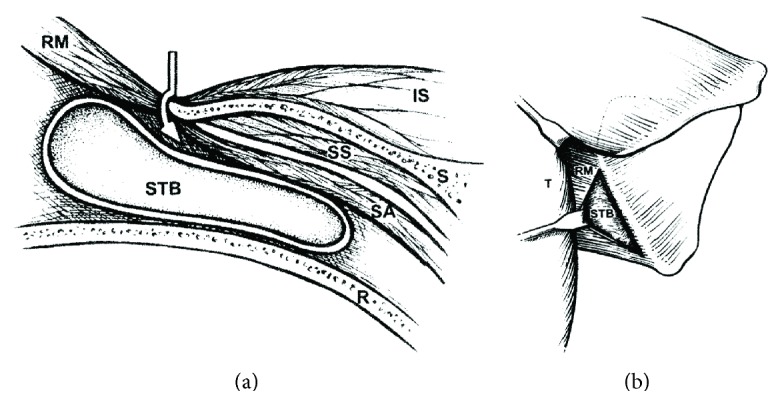
(a) Drawing of cross-sectional anatomy. The arrow shows the plane of dissection between the rhomboid major (RM) and the medial scapula (S), which allows access to the scapulothoracic bursa (STB). This bursa is in the space between the serratus anterior (SA) muscle and rib cage (R). IS, infraspinatus, (IS); SS, subscapularis. (b) Drawing of posterior view. The inferior trapezius (T) is retracted, exposing the rhomboid major (RM) muscle. The rhomboid major muscle is retracted, exposing the scapulothoracic bursa (STB), thus confirming the proper dissection plane (drawings reproduced from Nicholson and Duckworth (2002) with permission of Elsevier B.V.).

**Figure 3:**
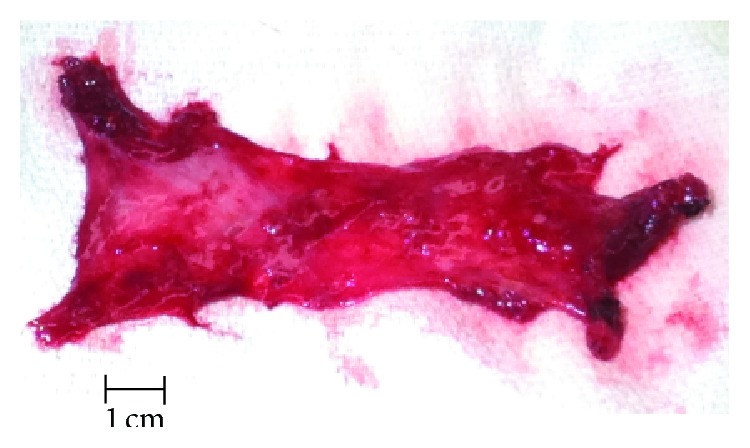
Tissue removed from the patient's right shoulder during the scapulothoracic bursectomy operation.

The patient was then tilted 30 degrees in the anterior direction for the open scapulothoracic bursectomy, which was done in accordance with the description in Nicholson and Duckworth [17] (Figure 2). Operative findings included a much thickened infraserratus bursa (Figure 3) with extensive transbursal adhesions extending from the mid-to-upper aspect of the medial scapula. The tendinous portion of the rhomboid major appeared thin, which was consistent with a traction tear [18]. Reattachment included transferring the insertion 1–1.5 cm onto the dorsal aspect of the scapula and repairing it with suture passed through a series of drill holes. During the deep surgical dissection the serratus anterior origin partially detached (approximately four cm in vertical length) and this was also repaired using the same drill holes. This problem, as described by Nicholson and Duckworth [17], is a known intraoperative complication of the dissection used to access the infraserratus space during the index surgery. Because intraoperative palpation revealed no bony abnormality, resection of the upper scapular angle was not done [13, 17, 19]. Notably, Nicholson and Duckworth [17] found that only five of their 17 patients required removing the superior-medial scapula.

The patient progressed in physical therapy in accordance with a protocol for the open anterior capsular shift procedure [16]. During the first six post-operative weeks, the headaches were gone. However, at that time the periscapular symptoms worsened and the headaches began recurring after a “popping sensation” was felt in the scapular region. This occurred when the patient was giving back massages in addition to performing unadvised lifting activities. The patient was then lost to follow-up for seven months. The patient then returned stating that the outcome of the shoulder surgery was good because it was “stable and gave me full function.” However, the headaches recurred and were triggered by activity-related pain and “grinding” in the mid region of the medial margin of the scapula.

**Figure 4:**
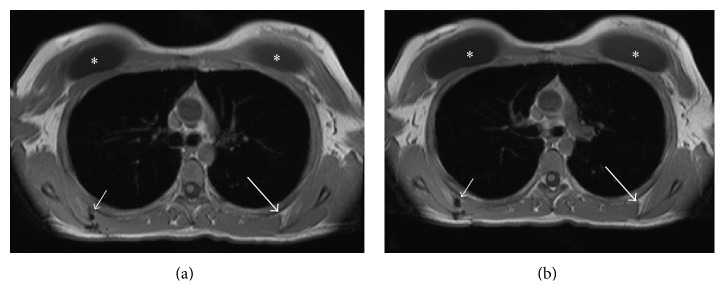
Magnetic resonance (MR) images of the patient's scapulothoracic regions ((a) is more cranial than (b)). The left side of each image shows the area of the injury of the patient's right scapulothoracic region (small arrows) and normal left side (larger arrows). The small arrows indicate recurrent bursitis and tearing of the serratus anterior and rhomboid attachments with disruption of suture material along the medial margin of the scapula. The asterisks indicate breast augmentation implants.

Physical examination showed scapulothoracic crepitus in the mid-scapular region but no winging. The right shoulder was globally stable and showed no limitations in strength or motion, and no other findings were detected. An MR scan of right and left scapulothoracic regions showed atypical tissue in the vicinity of the scapulothoracic bursa and nearby muscle attachments (Figure 4). This was consistent with torn muscle tissue and scar tissue at the site of the prior surgery.

A diagnostic local anesthetic injection (lidocaine and bupivacaine) was done in the region of the infraserratus bursa in this mid-scapular location. For one day this relieved the headaches and also gave 90% relief of the periscapular pain. Consequently revision surgery was then done. Operative findings included partial detachment (6 cm longitudinal length) of the serratus anterior and rhomboid major in the same location of the prior repair. This was associated with thickened bursa tissue, bursal adhesions, and torn sutures. There was also a scar-like band of tissue extending from this region upwards toward the superior scapular margin. This band and all abnormal tissue were excised. The insertion of the rhomboid major was reattached to the dorsal aspect of the medial scapula but a little farther (now 2–2.5 cm) to allow for more bone contact than at the index surgery (1–1.5 cm). Similar to the time of the index surgery, there was no bony abnormality and no clear reason to resect the superior-medial portion of the scapula. Microscopic evaluation of frozen and permanent sections of the tissue showed benign inflammatory tissue and no evidence of hemochromatosis/iron deposits [20]. The detached serratus was trimmed with scissors to produce a smooth margin and was then repaired to the ventral scapular surface and medial scapular margin using a double row of nonabsorbable sutures through drill holes (after gently burring the ventral scapular margin to produce punctuate bleeding). The medial row of drill holes was one centimeter from the medial scapular margin and the lateral row of drill holes was 2.5 centimeters from the medial scapular margin. The rhomboid muscle was also reattached to the dorsal scapula beneath the infraspinatus using sutures that were also placed through the medial row of drill holes prior to the serratus repair.

The patient was maintained in a sling for seven weeks with only elbow motion and passive shoulder motion allowed. Shoulder activity was then progressed with active-assisted motion from seven to 10 weeks after surgery. Resistive exercises were then progressed from that time until four months after surgery.

At the final follow-up, one year after revision surgery, the patient reported having excellent pain relief and restoration of function. The patient's preoperative ASES score (25) improved to 85 (0 = worst, 100 = best) [21]. Although the patient was able to perform some acrobatic exercises (e.g., the “skin the cat maneuver”), the patient did not return to performing the flagpole exercises because of a concern for recurrent injury.

## 3. Discussion

Scapular dyskinesis has been observed in patients with known glenohumeral joint pathology. For example, Warner et al. [8] reported this association in 14 of their 22 patients with shoulder instability and in all of their seven patients with subacromial impingement. In two other studies, scapular dyskinesis was observed in 15 out of 15 patients with glenohumeral instability [22, 23]. The 72 patients with scapular muscle detachment described by Kibler et al. [18] did not have glenohumeral instability. In contrast to all of these studies, our patient had a tear of the rhomboid major attachment, scapulothoracic bursitis, and glenohumeral instability that were simultaneously present and were caused by trauma during circus acrobatic maneuvers. In summary, the aspects of our case that make it unique include (1) the periscapular and glenohumeral pathologies occurred from unusual traumatic events, (2) the main traumatic event occurred during a flagpole exercise, (3) the patient was transgender (male-to-female) with pharmacological treatment with estradiol intramuscular injections for two years prior to the shoulder girdle trauma, (4) the headaches that were associated with these conditions improved after surgical correction of the scapulothoracic problems, (5) surgical correction of all shoulder girdle problems was done during the same operation, (6) a postoperative complication of tearing of the serratus anterior and rhomboid major attachments with recurrent scapulothoracic bursitis resulted from patient noncompliance and led to recurrent headaches, and (7) the postoperative complication was surgically corrected and ultimately resulted in an excellent outcome at one-year final follow-up.

In our review of the literature (PubMed and Google Scholar), we located very few reports of patients that had one or more of the abovementioned characteristics of our case. For example, six of 10 patients reported by Pavlik et al. [2] developed scapulothoracic bursitis after trauma. Of the six patients with trauma, four reported a fall onto the shoulder, one was injured in a motor vehicle accident, and one was injured defending at netball. One of these patients had glenohumeral symptoms which were associated with a SLAP lesion. Lehtinen et al. [11] described one patient (23-year-old male) who had a Bankart procedure prior to a scapulothoracic bursectomy that was done at a later date. But the authors did not provide sufficient information to determine if this patient sustained one traumatic event that had caused both conditions. In contrast to these two reports and our patient's case, when painful scapulothoracic bursitis with or without rhomboid muscle tearing is present these symptoms are usually not associated with glenohumeral instability or with other glenohumeral pathologies [3, 9, 11, 17, 18, 24, 25].

Recurrence of scapulothoracic bursitis following an open scapulothoracic bursectomy is uncommon. In the one case reported by Nicholson and Duckworth [17] the revision surgery required a partial scapulectomy (not done in our patient) but there was no serratus tear. Recurrence of scapulothoracic bursitis following partial scapulectomy has been reported in one case and was attributed to the development of excessive scar tissue [26]. Kibler et al. (2014) [18] reported that four of their patients that had repairs of serratus anterior tears and that two of these patients had traumatic retears. Posttraumatic tearing (without prior surgery) of the scapular attachment of the serratus anterior has been reported but is very rare [27–30]. These tears are typically associated with scapular winging. By contrast, our patient's serratus tear, which occurred in a weakened area of the muscle's origin, was not associated with winging presumably because it was comparatively small.

In our personal correspondence with Dr. W. B. Kibler, an authority on scapulothoracic dysfunction, we learned that he has a few patients (unpublished cases) with ipsilateral periscapular and glenohumeral pathologies that are similar to what we found in our patient. In these cases he has observed that the underlying problem has been the glenohumeral instability. In this context Dr. Kibler suggests that the snapping scapula/scapular bursitis in his patient (which is not associated with the scapular muscle detachment seen in our patient) results from the abnormal glenohumeral kinematics of the instability, and the inhibition of scapular stabilizing muscle activity also stems from the glenohumeral instability. Furthermore, Dr. Kibler has observed that the pectoralis minor and latissimus dorsi muscles become stiff/tight and the lower trapezius becomes weak, producing the abnormal scapular kinematics that led to excessive compression between the scapula and the underlying tissue. The ensuing scapular symptoms then become a significant part of the patient's dysfunction and limitations and can be resistant to physical therapy as long as the instability is present. With this concept of the pathophysiology, Dr. Kibler has treated the primary problem of the instability and has found that the scapulothoracic symptoms can usually be ameliorated with physical therapy once the pathoanatomy and altered muscle activation are optimized. Similarly, he and his colleague Dr. Jim Bradley have a series of patients that have posterior labral injuries and scapular dyskinesis and snapping and have treated all of them by repairing the labral lesion followed by physical therapy, which have produced very satisfactory results (unpublished findings). We provide this personal communication of these observations and unpublished cases so that surgeons are aware that it might be possible to avoid scapulothoracic surgery in some patients.

Additional aspects of our case that warrant consideration include the patient's diagnosis of hemochromatosis, the use of estradiol (to achieve and maintain transgender status) for two years prior to the shoulder girdle trauma, and the association of headaches with the periscapular symptoms.

Hereditary hemochromatosis is a disorder of iron metabolism in which there is excess iron absorption. Clinical manifestations of pathologic iron accumulation include liver disease, skin pigmentation, diabetes mellitus, cardiac enlargement, and joint osteoarthritis [31, 32]. Although hemochromatosis can be associated with joint pain, there is no reason to suspect that our patient's injuries or postoperative course were influenced by this condition. This is because hemochromatosis does not lead to joint laxity or to the increased propensity for joint injury.

There are data that indirectly support the possibility that our patient's two-year of estradiol use might have contributed to increasing the chance of injury that occurred during the flagpole exercises [33–36]. However, it is unclear if this was what occurred in our patient. For example, what tends to disfavor this hypothesis is that our patient did not have any other reports of other joint injuries despite being very physically active during the two years that estradiol was being taken.

There are reports of migraine-like headaches being associated with neck pain (upper trapezius) in ways that resemble, in part, the migration of our patient's headaches [18, 37–39]. The study of Kibler et al. [18] is the only one that we could locate that described in some detail the association of headaches with the presence of scapulothoracic bursitis or its surgical treatment. In their study of 72 patients with scapular muscle detachment (none with glenohumeral instability), 20–25% reported headaches as one of the major problems. These headaches typically arose from the upper trapezius and they all improved with muscle reattachment (personal communication, Dr. W. B. Kibler). Repairing the rhomboid major also appears to be the key step in successfully treating our patient's headaches.
